# Alzheimer's disease staging by instrumental activities of daily living (IADL): a crosswalk with the Montreal Cognitive Assessment (MoCA)

**DOI:** 10.1002/alz70857_107009

**Published:** 2025-12-25

**Authors:** Peter J Morin, Ying Wang, Joel Reisman, Dan Berlowitz, Brant Mittler, Raymond Zhang, Amir Abbas Tahami Monfared, Quanwu Zhang, Weiming Xia

**Affiliations:** ^1^ Boston University Chobanian & Avedisian School of Medicine, Boston, MA, USA; ^2^ Wentworth Institute of Technology, Boston, MA, USA; ^3^ University of Massachusetts Lowell, Lowell, MA, USA; ^4^ Geriatric Research Education & Clinical Center, VA South Texas Healthcare System, San Antonio, TX, USA; ^5^ Clinical Evidence Generation, Deep Human Biology Learning, Eisai Inc., Nutley, NJ, USA

## Abstract

**Background:**

Alzheimer's disease progression is typically staged using cognitive tests. This study aimed to define clinical stages measured by Instrumental Activities of Daily Living (IADL) using a crosswalk with Montreal Cognitive Assessment (MoCA) scores.

**Method:**

Patients with mild cognitive impairment (MCI) or Alzheimer's dementia (AD) were identified from the Veteran's Affairs Healthcare System (VAHS) database from 2020 – 2024. Paired MoCA and Lawton‐Brody IADL scores were analyzed to generate IADL score means, standard deviations (SD), medians, and ranges. Linear and repeated measures mixed effects analysis was performed adjusting for patient demographics.

**Result:**

The study sample (*N* = 1,327) had a mean age of 80.1 years (97.4% men, 18.4% Black, and 12.0% Hispanic). MoCA cut‐offs for Normal, MCI, Mild, Moderate, and Severe AD stages were ≥29, 26,18, 11 and ≤10, respectively. Corresponding mean (SD) and median IADL score cutoffs separating disease stages were: normal (*n* = 14), 7.0 (1.8) and 8; MCI (*n* = 96), 6.4 (2.3) and 8; mild (*n* = 666) 5.9 (2.5) and 7; moderate (*n* = 393) 4.0 (2.9) and 3; severe (*n* = 158), 2.4 (2.6) and 1. IADL tests resulted in ranges of 3‐8 for normal, 1‐8 for MCI, and 0‐8 for other stages. The IADL linear least squares (LS) means were distributed as >7.6, 6.9‐7.6, 5.1‐6.9, 3.5‐5.1, <3.5 for normal, MCI, mild, moderate, and severe stages projected by MoCA cutoffs, respectively (adjusted R‐squared=0.25). The LS means from categorical regression were 7, 6.5, 6.0, 4.2, and 2.6 for normal, MCI, mild, moderate and severe stages, respectively (adjusted R‐squared=0.23). Similarly, LS means from repeated measures analysis found IADL score ranges for normal to severe AD were >7.3, 6.7‐7.3, 5.1‐6.7, 3.6‐5.1, and <3.6.

**Conclusion:**

This study identified IADL score thresholds corresponding to clinical stages from normal to severe AD according to a regressional crosswalk with MoCA. The findings from categorial regression allowed greater spread of the IADL cut‐offs from mild to severe AD stages compared to linear and repeated measures regressions, accommodating nonlinearity in the cut‐off score distribution.

